# Design of polychromatic focusing optics for neutron reflectometers: rethinking REFocus optics

**DOI:** 10.1107/S1600576726002463

**Published:** 2026-05-14

**Authors:** Norifumi L. Yamada, Masahiro Hino, Ryuji Maruyama, Takuya Hosobata

**Affiliations:** a Institute of Materials Structure Science High Energy Accelerator Research Organization Tsukuba Ibaraki 305-0801 Japan; b Department of Materials Structure Science, The Graduate University for Advanced Studies (SOKENDAI), Tsukuba, Ibaraki305-0801, Japan; c Kyoto University Institute for Integrated Radiation and Nuclear Science Kumatori Osaka 590-0494 Japan; d J-PARC Center Japan Atomic Energy Agency 2-4 Shirakata Tokai Ibaraki 319-1195 Japan; e RIKEN Center for Advanced Photonics RIKEN Wako Saitama 351-0198 Japan; Technical University of Denmark, Denmark

**Keywords:** neutron reflectometry, focusing mirrors, wide-*Q* measurement, polychromatic beams, REFocus optics

## Abstract

Neutron reflectometry is a widely used non-destructive technique for analyzing surface and interfacial structures. In this study, we focused on REFocus optics, which employ an elliptical mirror to enhance measurement efficiency and extend the measurable *Q* range for a measurement with continuous neutron beams. The REFocus optics leverage a large beam divergence and polychromatic illumination to naturally provide angle and wavelength dispersions under specular reflection conditions. Using a position-sensitive detector simultaneously encodes both the dispersions into *Q* values, enabling highly efficient measurements compared with conventional methods. Here, we propose a compact REFocus optics design and demonstrate that combining a wide beam divergence with the novel concept of utilizing higher-order reflections covers a wide *Q* range from 0.08 to nearly 3 nm^−1^. Additionally, this approach yields a reduction in acquisition time of up to 100 times compared with conventional optics and offers practical advantages in cost and implementation. These findings provide a promising pathway for improving reflectometry performance at reactor sources.

## Abstract 2

REFocus optics, employing an elliptical mirror and higher-order reflections, enable rapid wide-*Q*-range neutron reflectometry at reactor sources, achieving up to 100-fold faster measurements compared with conventional methods.

## 1. Introduction

Neutron reflectometry is a widely used non-destructive technique for analyzing the structure of materials’ surfaces and interfaces. It enables the evaluation of scattering-length-density distributions at depths ranging from sub-nanometre to sub-micrometre scales. Though conceptually similar to X-ray reflectometry, neutron reflectometry leverages unique neutron properties, including high penetration power, isotope sensitivity and magnetic sensitivity. These features allow for the analysis of buried structures such as solid/liquid interfaces, selective visualization of components through deuteration labeling and evaluation of magnetic field distributions in magnetic materials, enabling more diverse and advanced measurements. Due to these characteristics, neutron reflectometry plays an important role in academic and industrial fields such as adhesion (Shiraki *et al.*, 2024 ▸), coating (Xia *et al.*, 2013 ▸), cleaning (Phan *et al.*, 2014 ▸), electrodes (Kawaura *et al.*, 2016 ▸) and magnetic thin films (Amemiya *et al.*, 2014 ▸). Its high demand has led to its establishment as an indispensable tool at neutron experimental facilities.

In neutron reflectometry, structural information at the sample surface and interface is primarily obtained by measuring reflectivity under specular reflection conditions. This measurement requires evaluating the dependence on the momentum transfer perpendicular to the reflecting surface, *Q* 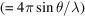, which requires scanning either the incident angle θ or the neutron wavelength λ. Facilities using steady-state beams, such as reactors, have widely adopted the angle-dispersive method, which measures reflectivity by varying θ using monochromatic neutrons (Bottyán *et al.*, 2013 ▸; Yuan *et al.*, 2011 ▸; Basu & Singh, 2006 ▸; Mattauch *et al.*, 2018 ▸; Georgii *et al.*, 2007 ▸; Lee *et al.*, 2015 ▸; Kwon *et al.*, 2007 ▸; Devishvili *et al.*, 2013 ▸; Yamazaki *et al.*, 2009 ▸). While this method is conceptually simple and provides high accuracy, it requires individual measurements at each angle, making it time consuming to cover a wide *Q* range. In contrast, the time-of-flight (TOF) method, which uses pulsed beams, allows a λ scan based on neutron velocity, enabling simultaneous measurement over a wide wavelength range. This method allows for the efficient acquisition of a broad *Q* range with fixed θ and is particularly useful for time-resolved measurements. Consequently, recent reactor-based instruments increasingly employ choppers to pulse the beam and apply the TOF method, although this approach has the drawback of reduced neutron flux per unit time (James *et al.*, 2011 ▸; Le Brun *et al.*, 2023 ▸; Kampmann *et al.*, 2007 ▸; Saerbeck *et al.*, 2018 ▸; Campbell *et al.*, 2011 ▸; Stahn & Glavic, 2016 ▸). Although TOF is widely recognized as useful, the reduction in neutron intensity due to pulsing remains a problem. One proposed solution is to measure wavelength dependence using a steady beam without pulsing. The RAINBOWS reflectometer, developed at the Institut Laue–Langevin in France, is the representative example. It reflects white neutrons from the sample, disperses λ using a prism and acquires spectra with a position-sensitive detector (PSD) (Cubitt *et al.*, 2018 ▸). Similarly, the CANDOR instrument, developed at the National Institute of Standards and Technology in the USA, employs an energy-dispersive detector with an array of analyzer crystals to simultaneously detect neutrons with different λ (Majkrzak *et al.*, 2019 ▸). These instruments achieve λ dispersion without pulsing the beam, thus avoiding intensity loss. On the other hand, the Amor reflectometer at the Paul Scherrer Institute in Switzerland employs a chopper to pulse the beam to use TOF with an elliptical mirror focusing optics, called the Selene guide, to compensate for the reduction in intensity caused by pulsing (Stahn & Glavic, 2016 ▸). Thus, there are multiple approaches to improve reflectivity measurements with steady beams, each employing different technical strategies to address challenges.

The REFocus concept proposed by Ott & Menelle (2008 ▸) demonstrates the potential of combining an elliptical focusing mirror with a graded multilayer coating to correlate wavelength and incidence angle in a continuous manner. However, its practical implementation poses several challenges, particularly the requirement for metre-scale elliptical optics and a spatially varying multilayer period. These features are central to the original idea, yet they naturally lead to demanding fabrication and installation conditions. In the present work, we revisit the REFocus principle from the perspective of practical deployment at a new 10 MW-class reactor that is being planned in Japan. We examined the applicability of the REFocus optics as part of efforts to improve reflectivity measurements with steady beams, aiming for compactness, mechanical simplicity and robustness. Instead of relying on a graded multilayer, we adopt a constant-period multilayer coating combined with the use of first- and third-order reflections to achieve a broad operational wavelength range. This approach preserves the fundamental angle–wavelength correlation intrinsic to an elliptical geometry while greatly simplifying fabrication. Furthermore, we examine geometric conditions under which the beam footprint remains nearly constant over a wide incidence-angle range by exploiting the compensation between optical magnification and grazing-incidence expansion. This aspect, which becomes critical for compact focusing systems, has not been discussed in detail in previous work. Through quantitative evaluation of the accessible *Q* range and reflected intensity, and comparison with conventional slit-collimated and TOF optics, we show that a compact REFocus-type instrument can retain the advantages of the original concept while offering improved feasibility for real-world implementation.

## 2. Design of REFocus optics

**Figure 1:**
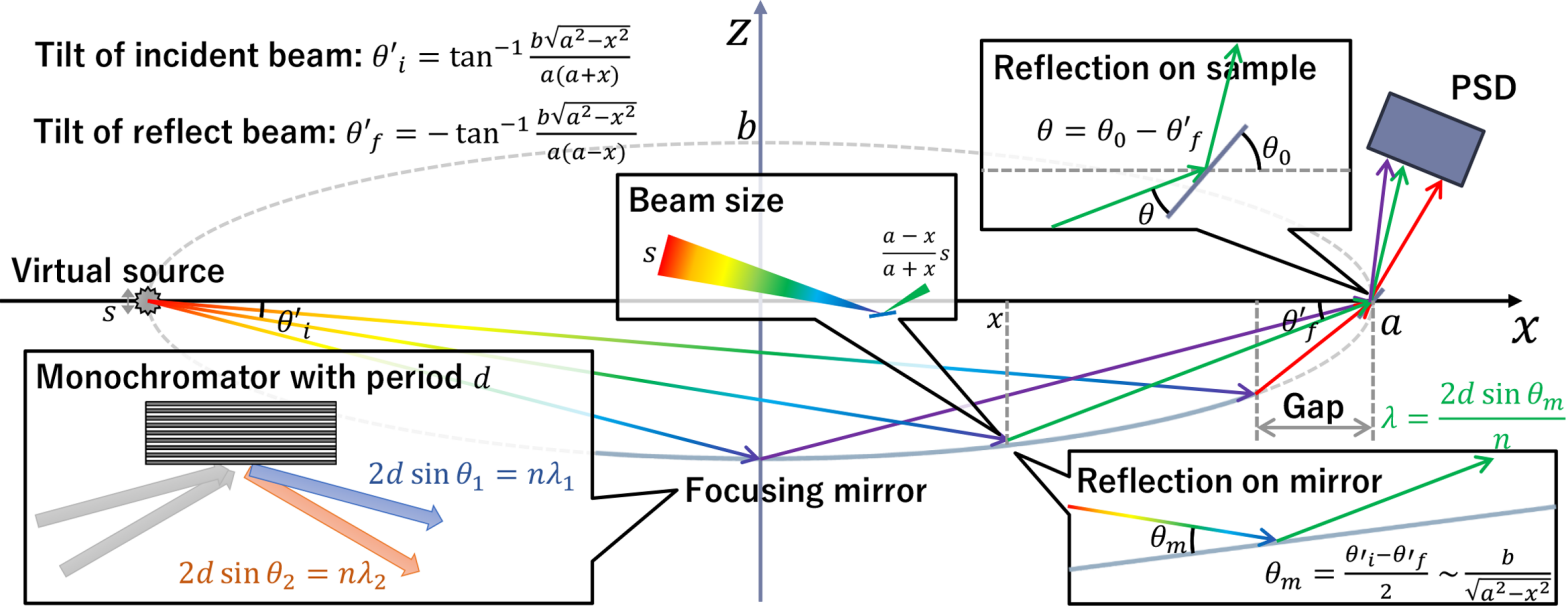
Design concept of REFocus optics (Ott & Menelle, 2008 ▸).

The design presented here follows the central optical principle of REFocus optics – namely, that a divergent source can be refocused onto the sample with an elliptical mirror, creating a natural correlation between incidence angle and wavelength (Ott & Menelle, 2008 ▸) (see Fig. 1 ▸). In this system, the axis passing through the virtual source and the sample is defined as the *x* axis, and the perpendicular direction is defined as the *z* axis. Neutrons emitted from the virtual source at an angle 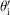 with respect to the *x* axis are reflected by a one-dimensional elliptical monochromatic mirror with a long axis *a* in the *x*-axis direction, a short axis *b* in the *z*-axis direction and a multi-layered coating of periodicity *d*. These neutrons are then focused onto the sample at an angle 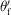 with respect to the *x* axis. Due to the variation in the incident angle on the mirror, θ_m_, depending on the *x* position, the wavelength λ satisfying the Bragg condition varies along each path: 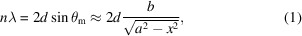
where *n* is the reflection order. Under specular reflection, the reflection angle is twice the incident angle θ at the sample – the difference between 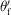 and the sample offset angle θ_0_ with respect to the *x* axis. Therefore, a PSD can encode both θ and λ, allowing conversion to *Q*, where the spatial resolution of the detector limits the resolution of θ. In contrast to the original realization, which employs a graded multilayer to extend the wavelength band along the mirror surface, a constant-period multilayer coating is used to keep the optics simple. To retain a wide wavelength acceptance without spatial grading of the multilayer, we exploit the coexistence of the first- and third-order Bragg reflections, enabling simultaneous measurement across a broad *Q* range.

An important consideration in this design is the beam footprint on the sample. The magnification of the elliptical mirror depends on the ratio of the distances from a reflection point on the mirror to the virtual source and the sample, which causes the beam size delivered to the sample to vary with the beam path. At the same time, because the beam impinges on the sample at a grazing angle θ, the illuminated footprint expands in inverse proportion to 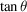. When the reflection directions at the mirror and the sample are aligned, these two effects counteract each other: larger incidence angles increase the projected beam size at the sample, whereas the expansion due to grazing incidence becomes weaker. For a virtual source of size *s*, the resulting footprint *l* can be described as 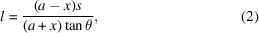
where (*a* − *x*)/(*a* + *x*) represents the effective magnification of the elliptical mirror for a beam reflected at position *x*. This compensation mechanism helps maintain the footprint within a limited area. Because a larger *s* increases the range of θ_m_ and thereby degrades the wavelength resolution, *s* is chosen such that the footprint remains below ∼10 mm in the present design. Although this geometry corresponds to an operational configuration similar to ‘Mode 2’ in the original REFocus paper (Ott & Menelle, 2008 ▸), the resulting stabilization of the footprint over a wide angular span has not been explicitly analyzed in previous work.

In addition to this geometric compensation, the following practical constraints were considered to optimize the optics, resulting in the parameters listed in Table 1 ▸:

(1) The minimum value of *Q* is set to 0.08 nm^−1^, which corresponds to 80% of the critical angle of the typical substrate, Si, used in reflectivity measurements.

(2) To cover a wider *Q* range, both first- and third-order reflections will be used.

(i) The second-order reflection at the monochromator mirror can be eliminated by using the extinction law.

(ii) To efficiently reflect the beam to obtain the third-order component, multilayer monochromator mirrors with a single period are stacked on a mirror with a period three times larger.

(3) Because Fresnel reflectivity decreases with decreasing λ, the center of the ellipse is set to satisfy the Bragg condition for the third-order reflection at the Maxwell peak (0.43 nm at 20 K in a liquid helium moderator), compensating for the low reflectivity with high flux.

(4) The mirror size and the period of the multilayer mirror are set to 400 mm and 9.75 nm (equivalent to 3*Q*_c_), respectively, considering availability and performance.

(5) To achieve a wide solid angle and sufficient space for installing sample environments, the distance between the downstream end of the focusing mirror and the sample is minimized to 150 mm.

These parameters enable the REFocus optics to focus a large-solid-angle beam of 92.6 mrad onto the sample within a compact 800 mm distance from the virtual source. Using both first- and third-order reflections, a broad *Q* range of 0.08–2.79 nm^−1^ can be measured with a wide wavelength band of 0.43–1.65 nm. This *Q* range is comparable to that achievable by multi-angle reflectometry at the SOFIA reflectometer in the Japan Proton Accelerator Research Complex (J-PARC) (currently under development by the authors; Yamada *et al.*, 2020 ▸), and to the measurable *Q* range for a typical sample. If switching between first- and third-order reflections can be performed rapidly, measurements on a similar timescale may be achievable at medium-sized reactors as well. In the next section, we detail the parameter-optimization process that led to this design.

## 3. Parameter optimization

**Figure 2:**
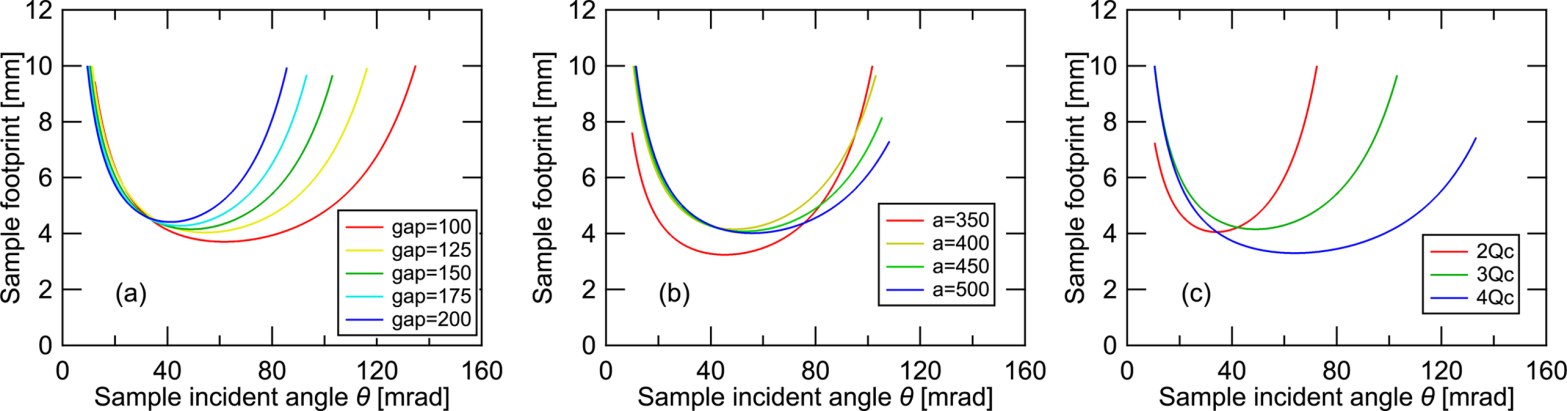
Beam footprint at the sample as a function of incident angle. (*a*) Effect of changing the mirror–sample gap. (*b*) Effect of changing the focal distance. (*c*) Effect of changing the multilayer period of the polychromator. The virtual-source size is adjusted so that the maximum footprint is ∼10 mm in all cases.

First, to evaluate the effect of each parameter on the beam footprint at the sample surface, we varied one optical parameter from Table 1 ▸ at a time and compared the results. Fig. 2 ▸ shows how the beam footprint changes with the incident angle θ when the gap between the mirror and sample, the focusing distance (distance between the two focal points), and the multilayer period are modified individually. As previously described, the footprint is determined by the balance between optical magnification along the beam path and expansion due to grazing incidence. Therefore, the footprint exhibits a minimum in the intermediate region of θ. In this study, the virtual source size was adjusted so that the footprint remained within 10 mm at all angles. As shown in Fig. 2 ▸(*a*), changing the gap distance does not significantly affect the footprint at low and high angles, confirming good matching in the design. In contrast, Figs. 2 ▸(*b*) and 2 ▸(*c*) show that changing the focusing distance or multilayer period significantly alters the footprint balance. If the footprint becomes too large at low angles, there is room to extend the mirror into the upstream side. However, perfect symmetry between low and high angles is not essential, and the final design prioritizes balance between the measurement intensity and *Q* range.

**Figure 3:**
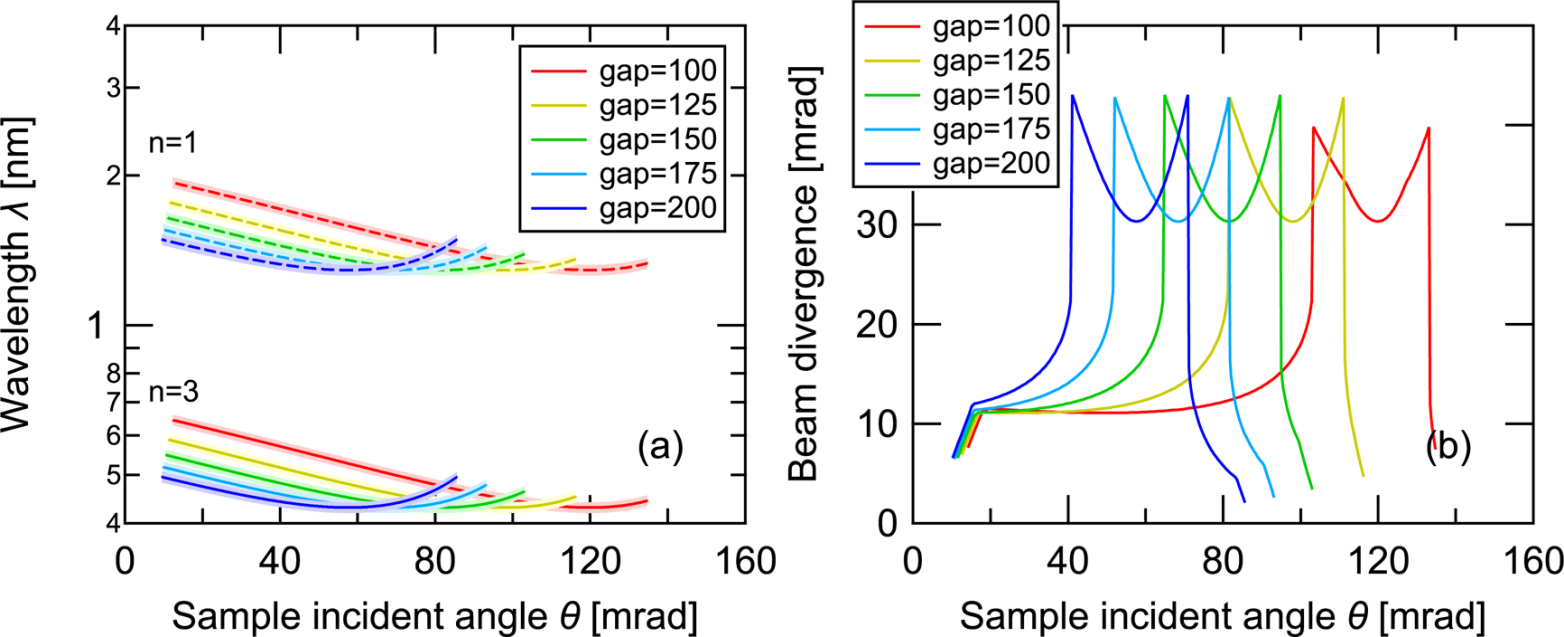
Relation between sample incident angle and (*a*) wavelength selected by the polychromator mirror when used together with the TOF method and (*b*) the resulting beam divergence for different mirror–sample gaps. Panel (*b*) is obtained by taking horizontal cuts at fixed wavelength from the shaded wavelength–angle map in panel (*a*), assuming a wavelength resolution of 5%. The angular width of each cut corresponds to the beam divergence plotted in (*b*).

**Figure 4:**
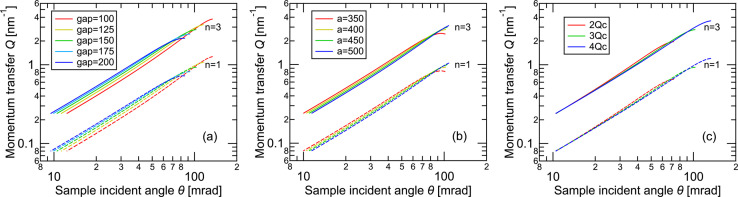
Incident-angle dependence of momentum transfer *Q*. (*a*) Effect of changing the mirror–sample gap. (*b*) Effect of changing the focal distance. (*c*) Effect of changing the multilayer period. Each condition corresponds to those used in Fig. 2 ▸.

Next, to evaluate the wavelength λ and momentum transfer *Q* range, which depend on the beam path, we varied the gap distance between the mirror and the sample again, as in Fig. 2 ▸(*a*). As shown in Fig. 3 ▸(*a*), λ changes with θ according to the Bragg condition of the monochromator mirror due to changes in the beam path as indicated by equation (1). As reflectivity as a function of *Q* will be analyzed in reflectometry, this relation will be used to convert the *Q* dependence on θ as shown in Fig. 4 ▸(*a*). Notably, the increase of *Q* becomes weaker (approaches an asymptote) at large θ, which results from the dependence of λ on θ: as λ increases with θ above the point at the center of the ellipse showing the λ minimum, the increase in *Q* is suppressed. Additionally, the *Q* range can be extended by using the third-order reflection because its associated λ is one-third that of the first-order reflection. The *Q* range strongly depends on the gap, because the solid angle of the mirror seen from the sample changes. In other words, a narrower gap leads to a wider distribution of incident angles at the sample, resulting in a broader *Q* range. Calculations of the *Q* ranges under the conditions shown in Figs. 2 ▸(*b*) and 2 ▸(*c*) are shown in Figs. 4 ▸(*b*) and 4 ▸(*c*), respectively. The *Q*range changed drastically with the multilayer period of the monochromator mirror, as shown in Fig. 4 ▸(*c*), due to the change in solid angle with respect to the gap. However, only a slight change in the *Q* range was observed when changing the focusing distance, with almost no change in beam divergence, as shown in Fig. 4 ▸(*b*). The change in Fig. 4 ▸(*b*) is due to the change in λ caused by the change in the incident angle of the mirror: a mirror with a longer focusing distance provides longer and shorter λ values at the lowest and highest θ values, respectively.

Though slightly outside the scope of parameter optimization, we note issues when using wide divergence optics, such as REFocus or Amor’s Selene guide. Classical reflectometry defines angular resolution by collimating a beam with a two-slit collimation system and measuring total reflected intensity to evaluate reflectivity. In contrast, wide-divergence optics assume specular reflection, meaning that the incident angle equals the reflection angle, and correct for this using a PSD (Cubitt *et al.*, 2015 ▸). However, rough surfaces exhibit off-specular reflection depending on in-plane structure, which contaminates the specular-reflection signal because diverged beams cannot separate the off-specular signal. Therefore, a method of separating the signals must be considered – this is a frequent topic of discussion regarding the use of large beam divergence. Since the monochromator mirror of REFocus optics works as a polychromator, λ varies with the beam path, that is θ. This suggests that an intensity map depending on θ and λ obtained by TOF could separate a specular reflection signal on the locus shown in Fig. 3 ▸(*a*) and off-specular reflection signal outside the locus. The key factor here is the wavelength resolution of the monochromator mirror, which is typically ∼5%, because it blurs the locus in the intensity map. Therefore, assuming a distribution of ±2.5% around the center value [as shaded in Fig. 3 ▸(*a*)], we calculated the beam divergence as a function of θ, shown in Fig. 3 ▸(*b*). The divergence is comparable to θ at the downstream side and the center of the mirror ellipse: it is around 10 mrad at small θ, gradually increases with θ, drastically increases up to 40 mrad around the local minimum of λ and drastically decreases at large θ. Although a monochromator with better λ resolution can reduce divergence, it cannot be suppressed at the ellipse center in principle because dλ/dθ is close to zero in this region. This means that the polychromator has insufficient resolution to separate the specular and off-specular signals, and a solution to this issue will be discussed later.

**Figure 5:**
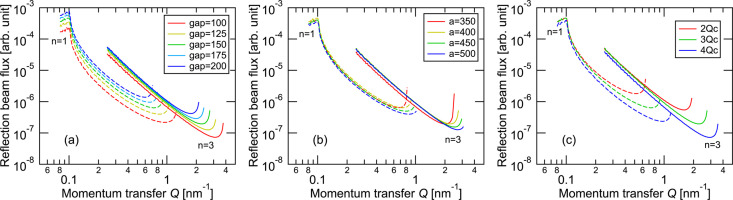
Reflected intensity as a function of *Q*. (*a*) Effect of changing the mirror–sample gap. (*b*) Effect of changing the focal distance. (*c*) Effect of changing the multilayer period. Each condition corresponds to those used in Fig. 2 ▸.

Finally, we will address the most important parameters: the *Q* range and the reflection intensity. The incident neutron beam intensity as a function of θ was calculated by dividing the mirror into fine segments, where the product of the virtual source size and the solid angle subtended by each segment from the source is proportional to the phase-space volume in the θ direction, that is, the beam flux transferred to the sample position via this segment. To account for the effect of λ on the flux, the Maxwell distribution at 20 K was used to evaluate the intensity distribution of a neutron beam as a function of wavelength as
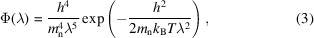
where *h* is Planck’s constant, *m*_n_ is the neutron mass, *k*_B_ is Boltzmann’s constant and *T* is the moderator temperature. To simplify the matter, the λ resolution was ignored, and the reflectivity of the monochromator was assumed to be 100%. The reflection intensity at each segment was calculated by multiplying the incident intensity by the reflectivity of a silicon substrate and binning them with a bin width of 2%. As shown in Fig. 5 ▸, for all optical parameters, first-order reflections exhibit a sharp decrease in reflectivity at the critical angle of total reflection. They then gradually decrease with increasing *Q*, reach a minimum at *Q* corresponding to the mirror center, and recover, consistent with the asymptotic behavior of *Q*, shown in Fig. 4 ▸. This improves measurement efficiency in higher-*Q* regions where intensity is usually weaker. Third-order reflections exhibit similar behavior, except for total reflection, and cover *Q* up to 2 nm^−1^ for all optical parameters. Using narrow gaps or 4*Q*_c_ monochromators can extend coverage beyond *Q* = 3.5 nm^−1^, but reflection intensity decreases as the *Q* range extends. Therefore, the parameters in Table 1 ▸ are adopted as optimal for intensity comparisons with other optics in the next section.

## 4. Comparison between optics

First, we examined comparisons with angle-dispersive and wavelength-dispersive methods using conventional two-slit collimation optics. To keep the overall system length similar to that of the REFocus optics, we set the sample-to-first-slit distance, *L*_1_, to 1000 mm, the sample-to-second-slit distance, *L*_2_, to 150 mm, and the beam footprint at the sample position, *l*, to 10 mm. Under these conditions, the slit widths *w*_1_ and *w*_2_ of the first and second slits, respectively, were determined by the following equations: 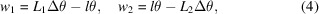
where the beam divergence, Δθ, satisfying 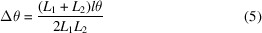
gives the maximum intensity because the product of *w*_1_ and *w*_2_ is maximized. Therefore, a beam divergence of 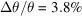, derived with *L*_1_ = 1000, *L*_2_ = 150 and *l* = 10, is employed in the following calculations. The phase-space volume was then calculated as *w*_1_*w*_2_/(*L*_1_ − *L*_2_), which allows for comparison with the reflected intensity of REFocus optics.

Regarding the neutron wavelength, the peak wavelength of the Maxwell distribution at 20 K (0.43 nm) was used for the angle-dispersive method, while the same wavelength range as the REFocus optics (0.43–1.65 nm) was used for the wavelength-dispersive method. The wavelength resolution was set to 5% standard deviation with peak reflectivity of 100% for all conditions, which was also considered for the REFocus optics. For the TOF method, the chopper duty ratio was determined to be 1.3% according to the following equation, ensuring a wavelength resolution of 5% at 0.43 nm: 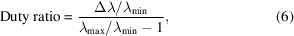
and the beam intensity was attenuated accordingly.

Additionally, the beam intensity of focusing optics when replacing the monochromator mirror in the original REFocus optics with a supermirror was evaluated to compare the advantages of a polychromatic continuous beam versus a pulsed beam using the TOF method. To simplify the process, the mirror parameters were kept as in Table 1 ▸ with a 9*Q*_c_ supermirror, matching the wavelength band and duty ratio with those of the two-slit collimation TOF system, and the supermirror reflectivity was assumed to be 100% up to the critical angle. The resulting REFocus optics intensities with the monochromator and supermirror were binned with a width of 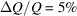, which is the same as for the TOF with the two-slit collimation optics for direct comparison. When the optics required a *Q* scan to cover the same *Q* range as the original REFocus, the intensity as a function of *Q* was normalized by dividing by the number of scan points.

**Figure 6:**
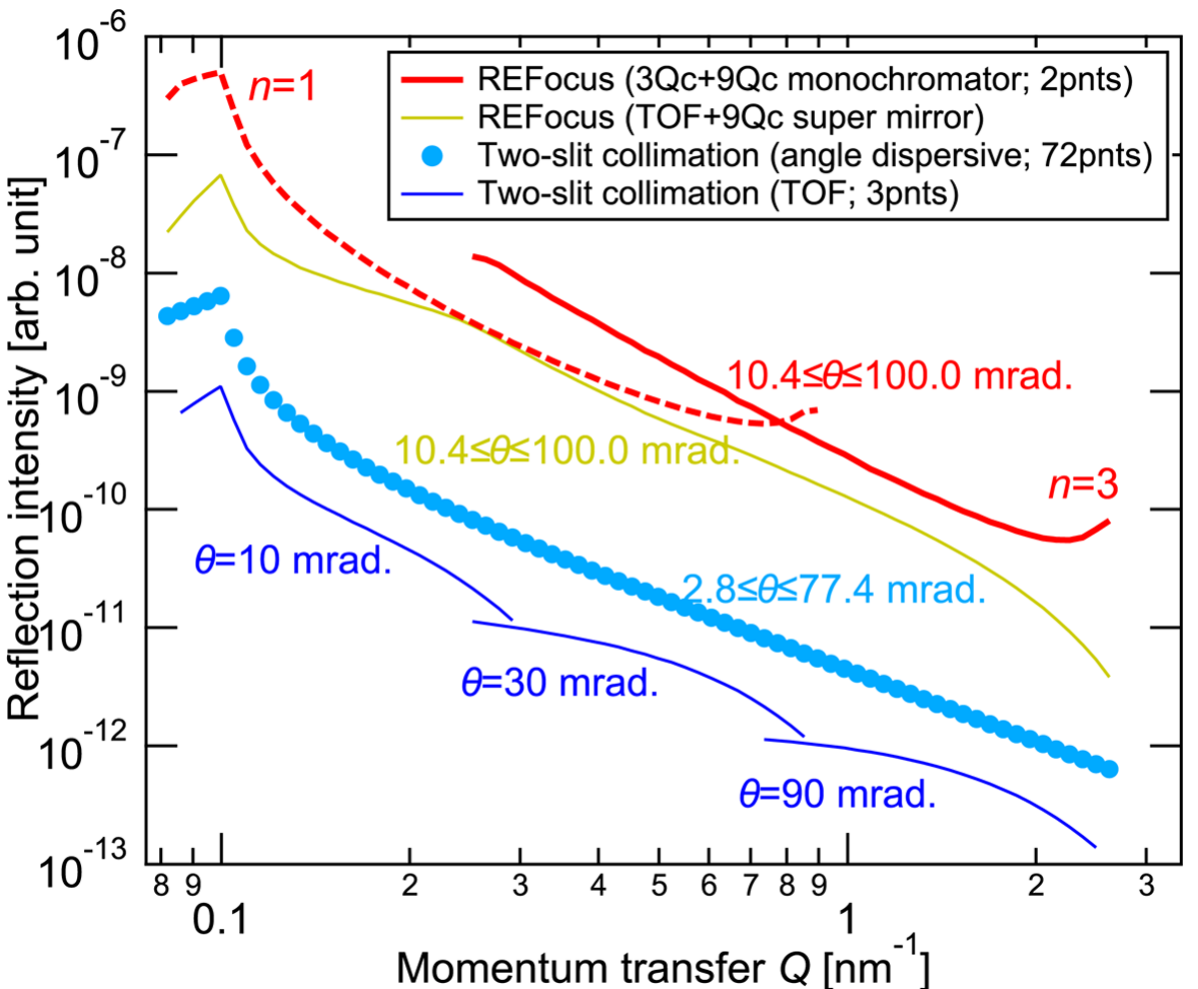
Comparison of reflected beam intensities between the proposed REFocus optics and conventional optics, as well as the REFocus system combined with the TOF method.

Fig. 6 ▸ shows the results of comparing the calculated reflection intensities under the above-described conditions. First, for the two-slit collimation optics, the reflected intensity obtained using the angle-dispersive method was ∼100 times weaker than that of the original REFocus optics, and the wavelength-dispersive method yielded an intensity several times lower than the angle-dispersive method. The two-orders-of-magnitude difference compared with the angle-dispersive method with two-slit collimation optics corresponds closely to the difference in the number of measurement points for the *Q* scan. This means that REFocus optics enable simultaneous acquisition across a wide *Q* range, resulting in a substantial gain. Furthermore, the REFocus optics offer a broader measurable *Q* range under a single measurement condition compared with the wavelength-dispersive method with the two-slit collimation optics, providing an inherent advantage. Additionally, using a continuous beam further increases the intensity of the original REFocus system. On the other hand, the footprint of ∼10 mm used in this study reflects an intrinsic optimization for the REFocus optics: a small virtual source size is required to maintain an adequate wavelength resolution when using a polychromator, and this constraint naturally limits the usable footprint. In contrast, slit-collimated optics can normally increase the footprint by widening the slits or using a larger sample, which increases the reflected intensity roughly in proportion to the footprint size. For the TOF methods, the divergence can be expanded even further because the PSD enables angle-resolved detection (Cubitt *et al.*, 2015 ▸), leading to an intensity increase that scales approximately with the square of the footprint. However, compensating for the gain achieved by the REFocus optics would require a footprint over 100 mm, which is generally impractical for typical reflectometry samples and sample environments.

Even when comparing the same focusing optics, the continuous beam with a monochromator mirror showed a higher intensity gain than the pulsed beam with a supermirror – despite the assumption of an ideal 9*Q*_c_ supermirror with 100% reflectivity, which is unrealistic. Notably, in the high-*Q* region, which typically limits measurement time, the original REFocus optics with a monochromatic beam achieved approximately tenfold gain. This result indicates that REFocus optics with a continuous beam and monochromatic mirror are more advantageous for steady-state neutron sources, such as reactors. On the other hand, the REFocus optics with a supermirror are expected to be more suitable for pulsed neutron sources, such as accelerator-based sources.

## 5. Implementation of REFocus

On the basis of the above considerations, REFocus optics show significant potential for improving the measurement efficiency of steady-state neutron sources compared with existing optics. However, several challenges are anticipated. First, one issue that has not been discussed is how to transport a highly divergent beam of 92.6 mrad. This is solved by installing a parabolic mirror upstream of the virtual source, which focuses only the nearly parallel component of the incoming beam. As a result, only rays with well defined and spatially ordered trajectories are delivered into the virtual source. This property becomes particularly advantageous when applying wavelength-selective filter mirrors. Because the elliptic monochromator functions as a polychromator, each ray path corresponds to a different effective Bragg wavelength determined by its incident angle on the elliptical surface. In principle, this requires the cutoff wavelength of each filter mirror to be matched to the ray path. However, the parallel-beam condition created by the parabolic mirror provides a natural spatial mapping between beam position and ray trajectory. Consequently, the local incidence angle – and therefore the critical wavelength – of the filter mirrors can be controlled precisely by adjusting their shape.

**Figure 7:**
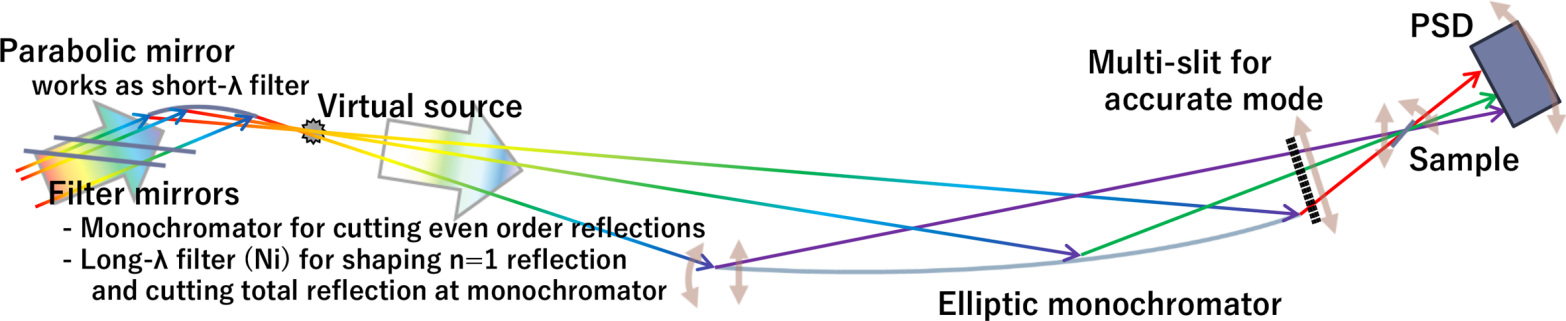
Instrument design proposal including devices to address the identified issues.

This makes the REFocus geometry remarkably well suited for efficient multi-stage filtering of unwanted spectral components via the following three filtering steps (Fig. 7 ▸):

(1) Short-wavelength suppression (fourth order and above): wavelengths shorter than 0.43 nm – corresponding to the fourth- and higher-order Bragg reflections of the elliptic monochromator – are removed by the parabolic mirror, which acts as a short-wavelength cutoff filter when its critical wavelength is set between the third- and fourth-order reflections.

(2) Second-order suppression: the even-order Bragg reflections of the elliptic monochromator are eliminated by an upstream multilayer monochromator whose first-order Bragg peak coincides with the second-order peak of the elliptic monochromator.

(3) Long-wavelength total-reflection suppression: neutrons with wavelengths above the critical angle of the elliptic monochromator undergo total reflection and generate an unwanted long-wavelength tail. This component is removed by a Ni mirror placed at a controlled incidence angle: wavelengths above the Ni critical angle are totally reflected out of the beam path, while the desired first- and third-order components are transmitted. Because the Ni cutoff can partially overlap with the first-order reflection band, this mirror also sharpens the wavelength distribution of the first-order component if needed. Furthermore, by adjusting the filter angle, the first-order component can be selectively removed, enabling clean switching between beams with and without the first-order contribution when scanning the *Q* range.

As a result of these three filtering processes, the beam entering the elliptic monochromator contains the useful first- and third-order components, but is free from second/higher-order or long-wavelength contamination.

Finally, a multi-slit at the elliptical mirror exit is considered to converge collimated beams with different angles for an accurate mode. This slit allows us to define the beam path from each slit, which detects not only the off-specular signal but also the broadening by the waviness of a sample that can be corrected under the assumption of specular reflection (Cubitt *et al.*, 2015 ▸). Although the multi-slit reduces the incident beam flux, it maintains an intensity more than ten times higher than conventional methods by maximizing the number of slit apertures. In cases requiring short acquisition times, such as time-slicing measurements to observe structural changes over time, the slit can be removed to conduct measurements in high-intensity mode. Fig. 7 ▸ shows the instrument design incorporating devices that address these issues.

## 6. Conclusions

In this study, we examined a compact reflectometer design with REFocus optics intended for reactor installation and compared it with conventional two-slit collimation optics. Table 2 ▸ summarizes the characteristics of reflectometer optics using steady beams. First, two-slit collimation optics are extremely simple and well established. Using monochromatic beams keeps costs low, but applying TOF for wavelength dispersion requires a chopper, which increases costs. Both methods allow for the separation of off-specular signals. Monochromatic beams provide higher accuracy since they are unaffected by refractive-index dependence on λ, while TOF efficiently measures a wide two-dimensional *Q* space. The *Q* range measurable in one measurement, without changing the incident angle, is extremely narrow for monochromatic beams but wide for TOF, whereas total acquisition time is shorter for monochromatic measurements. In contrast, REFocus optics enable the rapid scanning of a wide *Q* range by using a polychromatic beam with an extremely large divergence. The REFocus optics are not overly complex and contain no easily damaged components, suggesting low cost. Although the focusing mirror requires development, its short focal distance means that the required mirror precision is not excessively high. For example, focusing mirrors made of a metal substrate using ultra-precision machining have achieved a beam size of 0.3 mm at full width with a distance from the mirror to the sample of 2150 mm (Hosobata *et al.*, 2019 ▸; Yamada *et al.*, 2020 ▸). Since the effect of slope error on beam size is proportional to distance, a beam size of 0.02 mm can be achieved at minimum θ if a mirror with the same precision is used. Therefore, an elliptic mirror with lower precision is acceptable, as 0.02 mm at the minimum θ corresponds to a 2 mm footprint on the sample. One challenge involves handling off-specular reflection; however, adding a multi-slit immediately downstream of the mirror easily implements multi-incidence optics. This enables efficient measurements with collimated beams for separating off-specular signals. We considered using REFocus optics with TOF, but the intensity loss from the chopper outweighed the gain from wavelength dispersion, providing no advantage. However, this approach is expected to be beneficial for accelerator-based neutron sources with inherently pulsed beams.

At present, a new 10 MW-class reactor is being planned in Japan, and the reflectometer design suggested in this study, which uses REFocus optics, could be a suitable option for the actual instruments in the reactor. These optics offer numerous advantages in terms of both performance and practical aspects, such as cost, making them suitable for replacing existing reflectometers. These optics can also be adapted for pulsed sources, and their compact low-cost design is expected to be compatible with small accelerators, although some design modifications will be necessary – an issue for future work.

## Figures and Tables

**Table 1:** Optical parameters adopted in this study and the resulting measurable *Q* range Subscripts *n* = 1, 3 denote the Bragg reflection order.

Long axis	*a* = 400 mm
Short axis	*b* = 26.5 mm
Mirror position	−150 ≤ *x* ≤ 250 mm
Beam tilt to sample	44.7 ≤ θ′_f_ ≤ 137.3 mrad
Period	*d*_1_ = 9.75 nm (3*Q*_c_)
	*d*_3_ = 3.25 nm (9*Q*_c_)
Wavelength	λ_1_ = 1.29–1.65 nm
	λ_3_ = 0.43–0.549 nm
Sample offset angle	θ_0_ = 147.7 mrad
Momentum transfer	*Q*_1_ = 0.080–0.930 nm^−1^
	*Q*_3_ = 0.24–2.79 nm^−1^

**Table 2:** Comparison of optics for reflectometry with continuous beams

Optics	Wavelength selection	*Q* per point	Total acquisition time	Off-specular	Cost
Two-slit collimation	Monochromator	Very narrow	Long	Certain	Low
Two-slit collimation	TOF	Wide	Very long	Efficient	High
REFocus	Polychromator	Very wide	Very short	Undetectable	Possibly low
REFocus	TOF	Very wide	Short	Undetectable	High
REFocus and multi-slit	Polychromator	Very wide but sparse	Short	Detectable	Possibly low

## Data Availability

The data that support the findings of this study are available from the corresponding author upon reasonable request.
